# Self-assessed achievement of eating balanced meals – is it reliable for tailoring interventions?

**DOI:** 10.1093/eurpub/ckac131.238

**Published:** 2022-10-25

**Authors:** M Schneider, C Nössler, PM Lührmann

**Affiliations:** Institute of Health Science, University of Education, Schwäbisch Gmünd, Germany

## Abstract

**Background:**

Recording the self-assessed achievement of eating balanced meals offers the possibility to tailor nutritional interventions to the stage of behaviour change. Beforehand of an intervention, the self-assessed achievement of eating balanced meals was recorded and verified against the actual consumption.

**Methods:**

Self-assessed achievement of eating balanced meals was operationalised using the behavioral stages of the Health Action Process Approach (HAPA). The actual consumption was assessed by a validated 3-day dietary record and the Healthy Eating Index of the National Nutrition Survey (HEI-NVS) was calculated. A score of 100 points represents one hundred percent compliance with the recommendations of the German Nutrition Society. Ten additional points could be scored if vegetables and fruit were consumed above the recommendation. An HEI-NVS score of ≥ 80.0 points was considered a cut-off for ‘eating balanced meals'.

**Results:**

In a sample of 130 participants (86.9 % female, 29.0 ± 11.3 years), 9.2 % rated themselves as Non-Intenders, 17.7 % as Intenders, and 73,1 % as Actors. Their HEI-NVS was 69.6 ± 10.6 points, 79.2 ± 9.5 points, and 79.7 ± 9,0 points, respectively (ANOVA, p < 0.01). In the post-hoc-Test (Scheffé) Non-Inteders differed from the other groups (p < 0.05), Intenders and Actors were not different (n.s.). The proportion of participants with an HEI-NVS score ≥ 80.0 points was 16.7% for Non-Intenders, 60.9% for Intenders, and 51.6% for Actors (Chi^2^-Test, p < 0.05).

**Conclusions:**

Self-assessed achievement of eating balanced meals is characterised by self-underestimation (predominatly Intenders) and self-overestimation (predominatly Actors). Tailored interventions should take this into account.

**Key messages:**

• Self-assessed achievement of eating balanced meals is characterised by self-overestimation.

• Self-assessed achievement of eating balanced meals is also characterised by self-underestimation.

